# The effect of methotrexate and targeted immunosuppression on humoral and cellular immune responses to the COVID-19 vaccine BNT162b2: a cohort study

**DOI:** 10.1016/S2665-9913(21)00212-5

**Published:** 2021-07-08

**Authors:** Satveer K Mahil, Katie Bechman, Antony Raharja, Clara Domingo-Vila, David Baudry, Matthew A Brown, Andrew P Cope, Tejus Dasandi, Carl Graham, Thomas Lechmere, Michael H Malim, Freya Meynell, Emily Pollock, Jeffery Seow, Kamila Sychowska, Jonathan N Barker, Sam Norton, James B Galloway, Katie J Doores, Timothy I M Tree, Catherine H Smith

**Affiliations:** a St John's Institute of Dermatology, Guy's and St Thomas' NHS Foundation Trust, King's College London, London, UK; b Centre for Rheumatic Diseases, King's College London, London, UK; c Department of Immunobiology, Faculty of Life Sciences & Medicine, King's College London, London, UK; d Department of Infectious Diseases, School of Immunology and Microbial Sciences, King's College London, London, UK; e Psychology Department, Institute for Psychiatry Psychology and Neuroscience, King's College London, London, UK

## Abstract

**Background:**

Patients on therapeutic immunosuppressants for immune-mediated inflammatory diseases were excluded from COVID-19 vaccine trials. We therefore aimed to evaluate humoral and cellular immune responses to COVID-19 vaccine BNT162b2 (Pfizer-BioNTech) in patients taking methotrexate and commonly used targeted biological therapies, compared with healthy controls. Given the roll-out of extended interval vaccination programmes to maximise population coverage, we present findings after the first dose.

**Methods:**

In this cohort study, we recruited consecutive patients with a dermatologist-confirmed diagnosis of psoriasis who were receiving methotrexate or targeted biological monotherapy (tumour necrosis factor [TNF] inhibitors, interleukin [IL]-17 inhibitors, or IL-23 inhibitors) from a specialist psoriasis centre serving London and South East England. Consecutive volunteers without psoriasis and not receiving systemic immunosuppression who presented for vaccination at Guy's and St Thomas' NHS Foundation Trust (London, UK) were included as the healthy control cohort. All participants had to be eligible to receive the BNT162b2 vaccine. Immunogenicity was evaluated immediately before and on day 28 (±2 days) after vaccination. The primary outcomes were humoral immunity to the SARS-CoV-2 spike glycoprotein, defined as neutralising antibody responses to wild-type SARS-CoV-2, and spike-specific T-cell responses (including interferon-γ, IL-2, and IL-21) 28 days after vaccination.

**Findings:**

Between Jan 14 and April 4, 2021, 84 patients with psoriasis (17 on methotrexate, 27 on TNF inhibitors, 15 on IL-17 inhibitors, and 25 on IL-23 inhibitors) and 17 healthy controls were included. The study population had a median age of 43 years (IQR 31–52), with 56 (55%) males, 45 (45%) females, and 85 (84%) participants of White ethnicity. Seroconversion rates were lower in patients receiving immunosuppressants (60 [78%; 95% CI 67–87] of 77) than in controls (17 [100%; 80–100] of 17), with the lowest rate in those receiving methotrexate (seven [47%; 21–73] of 15). Neutralising activity against wild-type SARS-CoV-2 was significantly lower in patients receiving methotrexate (median 50% inhibitory dilution 129 [IQR 40–236]) than in controls (317 [213–487], p=0·0032), but was preserved in those receiving targeted biologics (269 [141–418]). Neutralising titres against the B.1.1.7 variant were similarly low in all participants. Cellular immune responses were induced in all groups, and were not attenuated in patients receiving methotrexate or targeted biologics compared with controls.

**Interpretation:**

Functional humoral immunity to a single dose of BNT162b2 is impaired by methotrexate but not by targeted biologics, whereas cellular responses are preserved. Seroconversion alone might not adequately reflect vaccine immunogenicity in individuals with immune-mediated inflammatory diseases receiving therapeutic immunosuppression. Real-world pharmacovigilance studies will determine how these findings reflect clinical effectiveness.

**Funding:**

UK National Institute for Health Research.

## Introduction

Immune-mediated inflammatory diseases including psoriasis, rheumatoid arthritis, and inflammatory bowel disease collectively affect 3–7% of European and North American populations.^1^, ^2^, ^3^ Drugs such as methotrexate, and biologics targeting the cytokines tumour necrosis factor (TNF), interleukin (IL)-17, and IL-23, are highly effective at attenuating the shared pathogenic immune pathways across immune-mediated inflammatory diseases, but they also increase the risk of serious infections, particularly with respiratory pathogens.^4^, ^5^ Notably, in many countries, including the UK, individuals with immune-mediated inflammatory diseases receiving therapeutic immunosuppressants were advised to undertake stringent public health risk-mitigating measures (shielding) early in the pandemic due to concerns over drug-related risks of severe illness from COVID-19.^6^ Shielding has led to reduced natural acquisition of protective immunity to SARS-CoV-2, and substantial psychological, social, and economic costs.

**Research in context**

**Evidence before this study**

Individuals with immune-mediated inflammatory diseases receiving therapeutic immunosuppression were excluded from COVID-19 vaccine trials; therefore, characterisation of vaccine efficacy in this vulnerable population is important. Given the roll-out of extended interval vaccination programmes, the effect of immunosuppression on the immunogenicity of a single dose of vaccine is of major public, and personal, health importance. Methotrexate, but not targeted biological therapies, can impair serological responses to influenza and pneumococcal vaccines, but whether these drug-specific effects can be generalised to COVID-19 vaccines is not known. We searched PubMed for peer-reviewed studies published up to April 16, 2021, using the terms “immunosuppression”, “COVID-19”, “vaccination immune response”, and “vaccine immunogenicity”, with no language restrictions. Studies on both humoral and cellular immunogenicity of the COVID-19 vaccine in patients with immune-mediated inflammatory diseases receiving immunosuppression had not been done. Early evidence relating to a range of cancer therapies suggests that both humoral and cellular responses to the first dose of the COVID-19 vaccine BNT162b2 (Pfizer-BioNTech) are severely attenuated.

**Added value of this study**

We evaluated the effect of methotrexate and biologics targeting tumour necrosis factor, interleukin (IL)-17, and IL-23 on humoral and cellular immune responses to the first dose of the COVID-19 vaccine BNT162b2 in patients with psoriasis. Seroconversion rates were lower in patients receiving immunosuppressants than in healthy controls, with the lowest rate identified in those receiving methotrexate. Neutralising titres against wild-type SARS-CoV-2 were also attenuated in patients receiving methotrexate compared with healthy controls but were preserved in those receiving targeted biologics. Neutralising activity against the B.1.1.7 variant was uniformly low among all participants, including healthy controls. By contrast, spike-specific T-cell responses (including interferon-γ, IL-2, and IL-21 production) were induced in patients receiving methotrexate and all classes of biologics, and were detectable at similar levels to those in healthy controls.

**Implications of all the available evidence**

These findings indicate that seroconversion alone might not adequately reflect vaccine immunogenicity in individuals with immune-mediated inflammatory diseases receiving therapeutic immunosuppression, and caution against routine use of seroconversion data in isolation in clinical practice. When taking into account functional humoral immunity and T-cell responses, our data suggest that targeted biologics do not impair vaccine responses and provide some reassurance to this vulnerable population. Notably, although methotrexate attenuated humoral immunity, cellular responses were preserved. Real-world pharmacovigilance studies will determine how these data reflect clinical effectiveness. Findings in relation to the B.1.1.7 variant indicate that those on immunosuppression, in common with the general population, might remain vulnerable after the first dose of BNT162b2, and therefore risk mitigation (in addition to vaccination) remains a key strategy in the pandemic.

Although mass vaccination promises a return to normality, there is a paucity of data on vaccine immunogenicity in patients with immune-mediated inflammatory diseases receiving immunosuppressants because they were excluded from trials of COVID-19 vaccines, including the now widely used BNT162b2 (Pfizer-BioNTech) vaccine. This vaccine comprises a lipid-nanoparticle-formulated nucleoside-modified mRNA encoding a full-length, stabilised SARS-CoV-2 spike glycoprotein.^7^ Studies evaluating immunogenicity to influenza and pneumococcal vaccines indicate that methotrexate, but not targeted therapies such as TNF inhibitors, impairs humoral responses, based on lower antibody titres in patients receiving this drug than in healthy controls.^8^ Successful host protection induced by vaccinations is mediated by a complex interplay between innate, humoral, and cellular immunity, all of which are likely to be required for effective and enduring defence against SARS-CoV-2.^9^ Thus, there is an urgent need to determine both functional antibody and antigen-specific cell-mediated immunogenicity to COVID-19 vaccines in individuals receiving different types of therapeutic immunosuppression to inform and, if necessary, revisit public health policy decisions. Assessment of immune responses to a single dose of vaccine is particularly pertinent given the roll-out of extended-interval vaccination programmes in countries such as the UK to maximise population coverage.

We studied patients with the immune-mediated inflammatory disease psoriasis to address this gap in knowledge, since therapeutic immunosuppression is generally prescribed as monotherapy, without concurrent oral corticosteroids.^10^ We aimed to investigate the effect of monotherapy with methotrexate, and biologics targeting TNF, IL-17, and IL-23, on immunogenicity after the first dose of BNT162b2. We sought to characterise both humoral and cellular immune responses.

## Methods

### Study design and participants

In this cohort study, we screened consecutive patients who presented to a specialist psoriasis centre (Severe Psoriasis Service, St John's Institute of Dermatology, Guy's and St Thomas' NHS Foundation Trust, London, UK) serving London and South East England. To be included in the study, patients had to have a dermatologist-confirmed diagnosis of psoriasis and had to be established on monotherapy with one of the following immunosuppressants: methotrexate, a TNF inhibitor (adalimumab, infliximab, etanercept, or certolizumab), an IL-17 inhibitor (ixekizumab or secukinumab), or an IL-23 inhibitor (guselkumab, risankizumab, or ustekinumab). Volunteers who did not have a diagnosis of psoriasis and were not receiving immunosuppressant drugs were also recruited as a healthy control cohort. All participants had to be eligible to receive the BNT162b2 vaccine. Given the role of previous infection on priming vaccine responses^11^ and the reported variation in intensity of cellular responses observed with different COVID-19 vaccines,^12^ individuals with past SARS-CoV-2 infection or those who received the ChAdOx1 nCoV-19 vaccine were excluded from the main analysis.

This study was approved by the London Bridge Research Ethics Committee (REC reference 06/Q0704/18). All participants provided written informed consent before enrolment.

### Procedures

Clinical and safety data and blood samples were collected at three study visits: baseline (same day of and immediately before vaccination), 28 days (±2 days) after the first dose of BNT162b2, and 14 days (±2 days) after the second dose (which was planned to be administered 12 weeks after the first dose as per UK Government guidelines). Data on immune responses after the second vaccine dose will be reported elsewhere.

Clinical and safety data included demographics, comorbidities (including psoriatic arthritis), details of psoriasis (including disease severity measured by the Psoriasis Area and Severity Index^13^), and local or systemic adverse events after vaccination. C-reactive protein was not assessed in any participants at the study visits. Plasma and peripheral blood mononuclear cells were isolated from blood samples as previously described.^14^ Details of immunogenicity assays are presented in the appendix (pp 2–3). Humoral immunogenicity was based on seroconversion, assessed using ELISAs for IgG specific for the SARS-CoV-2 spike glycoprotein, and the functional capacity of participants' plasma to neutralise both the prototypic (wild-type) strain of SARS-CoV-2 and the B.1.1.7 variant (VOC 202012/01 lineage), which has become highly prevalent in the UK following its emergence in late 2020. We first identified and excluded individuals with evidence of previous COVID-19 based on baseline IgG reactivity to both the SARS-CoV-2 nucleoprotein and spike protein.

To assess cellular immune responses to BNT162b2, we quantified interferon-γ, IL-2, and IL-21 responses to stimulation with two peptide pools spanning the entire length of the SARS-CoV-2 spike glycoprotein to ensure we captured a wider range of vaccine-induced responses than would be possible by detecting a single cytokine. Receiver operator characteristic curve analysis was used to establish threshold values to determine a positive response for the total T-cell response (combined interferon-γ, IL-2, and IL-21) and for each individual cytokine. Cytokine responses to peptides derived from commonly encountered viruses and fungal antigens were also assessed as controls. Given the association between T helper (Th) 17 cell activation and severe COVID-19,^15^, ^16^ we also analysed IL-17A and IL-22 responses to total spike peptide pools. Direct FluoroSpot assays were used to detect interferon-γ and IL-2, and IL-17A and IL-22 (Th17 cytokine profile), and direct ELISpot assays were used to detect IL-21.

### Outcomes

The primary outcomes were humoral and cellular immunity to the SARS-CoV-2 spike glycoprotein 28 days (±2 days) after the first dose of BNT162b2. Specifically, humoral immunity was defined as the presence of antibodies with neutralising capacity against the spike glycoprotein of wild-type SARS-CoV-2, and cellular immunity was defined as the presence of T cells secreting interferon-γ, IL-2, or IL-21 in response to stimulation with two peptide pools spanning the entire length of the SARS-CoV-2 spike glycoprotein.

Secondary outcomes (all of which were measured 28 days (±2 days) after the first dose of BNT162b2) were antibody seroconversion rates (presence of IgG antibodies specific for the spike glycoprotein, without assessment of neutralising capacity); the presence of antibodies with neutralising capacity against the spike glycoprotein of the SARS-CoV-2 pseudotype expressing the spike B.1.1.7 variant; the presence of T cells secreting IL-17A and IL-22 in response to stimulation with two peptide pools spanning the entire length of the spike glycoprotein; safety (adverse events); and patient-reported worsening of psoriasis.

Adverse events were classified as local or systemic, and graded as mild (no or minimal interference with activity and no or minimal medical intervention required), moderate (limitation in activity and medical intervention required), severe (marked limitation in activity and medical intervention required).

Results following the second dose will be presented elsewhere, and the primary and secondary outcomes will remain the same as the current study (but related to the second dose).

### Statistical analysis

Demographic and clinical characteristics, adverse events, and humoral and cellular immunogenicity were summarised using descriptive statistics. Statistical imbalance across the treatment groups was calculated with the Kruskal-Wallis or χ^2^ test. The key exposure measure was immunosuppressive treatment type, comprising five mutually exclusive groups: patients receiving methotrexate, TNF inhibitors, IL-17 inhibitors, or IL-23 inhibitors, and healthy controls (individuals not receiving immunosuppressive drugs).

We tested two pre-determined hypotheses: that patients taking methotrexate would have lower humoral, cellular, or both humoral and cellular immunogenicity to the first dose of BNT162b2 compared with healthy controls; and that patients taking methotrexate would have lower humoral, cellular, or both humoral and cellular immunogenicity to the first dose of BNT162b2 compared with patients taking targeted biological therapy (TNF inhibitors, IL-17 inhibitors, or IL-23 inhibitors).

Comparisons were made using linear regression of log-transformed immunogenicity measures, with robust estimates of variance to derive 95% CIs and p values. A 5% α level was used for significance testing. No correction for multiple hypothesis testing was made. Correlations were derived using Pearson's method. Data were analysed in Stata (version 16).

### Role of the funding source

The funder of the study had no role in study design, data collection, data analysis, data interpretation, or writing of the manuscript.

## Results

**Figure 1:**
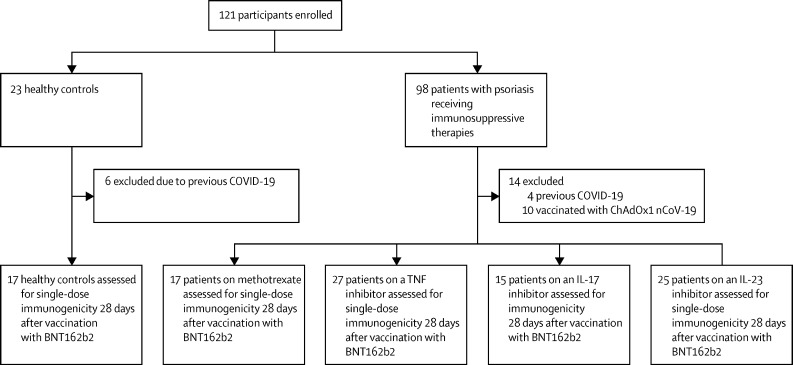
Study overview IL=interleukin. TNF=tumour necrosis factor.

Between Jan 14 and April 4, 2021, 98 patients with psoriasis receiving immunosuppressive drugs and 23 healthy volunteers (the control group) were recruited. We excluded ten participants (six controls, one patient on methotrexate, and three patients on an IL-23 inhibitor) from the final analysis on the basis of previous COVID-19 (appendix p 8) and ten individuals because they had been immunised with the ChAdOx1 nCoV-19 vaccine. The final analysis included 17 controls and 84 patients with psoriasis receiving monotherapy with methotrexate (17 [20%] patients), TNF inhibitors (27 [32%]), IL-17 inhibitors (15 [18%]), or IL-23 inhibitors (25 [30%]), all without concurrent glucocorticoids (figure 1). All participants included in the analysis followed the study schedule protocol.

The median age of participants was 43 years (IQR 31–52), 56 (55%) participants were male, 45 (45%) were female, and 85 (84%) were of White ethnicity (table 1). Among the 84 participants with psoriasis, the median duration of psoriasis was 21 years (IQR 15–33), 20 (24%) had concurrent psoriatic arthritis, and the median body-mass index was 28·7 kg/m^2^ (IQR 26·4–33·1). Adherence to immunosuppression therapy was confirmed with all participants, and no participants reported pausing or stopping their immunosuppressants before or during vaccination. The median dose of methotrexate was 15 mg (IQR 15–20) per week. The median duration of the current immunosuppressive treatment was 3·3 years (IQR 1·3–5·3), and participants in all study groups had well controlled psoriasis, as indicated by their baseline disease severity. Baseline characteristics disaggregated by sex are shown in the appendix (p 4).

**Table 1:** Baseline characteristics of study participants

		**Healthy controls (n=17)**	**Patients on methotrexate (n=17)**	**Patients on TNF inhibitors (n=27)**	**Patients on IL-17 inhibitors (n=15)**	**Patients on IL-23 inhibitors (n=25)**	**Total (n=101)**	**p value**
Age, years		34·0 (27·0–46·0)	48·0 (41·0–56·0)	36·0 (28·0–52·0)	45·0 (38·0–49·0)	50·0 (33·0–56·0)	43·0 (31·0–52·0)	0·053*
Sex		..	..	..	..	..	..	0·84†
	Male	9 (53%)	11 (65%)	13 (48%)	8 (53%)	15 (60%)	56 (55%)	..
	Female	8 (47%)	6 (35%)	14 (52%)	7 (47%)	10 (40%)	45 (45%)	..
Body-mass index, kg/m^2^		23·0 (21·5–30·5)	27·1 (26·4–29·7)	31·2 (26·2–39·4)	27·6 (25·1–30·9)	28·7 (27·0–33·1)	28·2 (25·1–32·7)	0·032*
Ethnicity		..	..	..	..	..	..	0·72*
	White	14 (82%)	13 (76%)	24 (89%)	13 (87%)	21 (84%)	85 (84%)	..
	Black	0	1 (6%)	0	0	0	1 (1%)	..
	South Asian	3 (18%)	3 (18%)	3 (11%)	2 (13%)	3 (12%)	14 (14%)	..
	Mixed	0	0	0	0	1 (4%)	1 (1%)	..
Disease severity measure (Psoriasis Area Severity Index^13^)		..	2·2 (1·3–3·5)	1·1 (0·6–2)	0·6 (0–1·8)	1·2 (0–2·8)	1·2 (0·6–2·8)‡	0·11†
Concomitant psoriatic arthritis		..	2 (12%)	5 (19%)	8 (53%)	5 (20%)	20/84 (24%)	0·027*

Data are median (IQR), n (%), or n/N (%) unless otherwise specified. Statistical imbalance of the baseline characteristics across the treatment groups is presented by either the Kruskal-Wallis or χ^2^ test. IL=interleukin. TNF=tumour necrosis factor.

* χ^2^ test.

† Kruskal-Wallis test.

‡ Median for the 84 patients with psoriasis.

63 (75%) of 84 patients with psoriasis receiving immunosuppression therapy reported mild adverse events within 28 days after the first dose of the BNT162b2 vaccine, compared with 16 (94%) of 17 controls (appendix p 6). Adverse event data were missing for four patients. 17 (20%) patients and one (6%) healthy control reported no adverse effects, and no participants reported moderate or severe adverse events. The commonest local adverse effect was pain at the injection site, which was reported by 55 (65%) patients and 14 (82%) controls. Systemic adverse effects were reported by 36 (43%) patients and seven (41%) controls. The commonest systemic adverse events were fatigue and headache. No participants reported COVID-19 during the study. Nine (11%) patients reported that their psoriasis worsened after vaccination: four were on IL-23 inhibitors, two on methotrexate, and three on TNF inhibitors.

Humoral and cellular data were missing for two patients on methotrexate, three patients on TNF inhibitors, and two patients on IL-23 inhibitors. No cellular data were available for one healthy control. In the control group, 17 [100%; 95% CI 80 to 100) of 17 participants had evidence of seroconversion after vaccination with BNT162b2, compared with 60 (78%; 67 to 87) of 77 patients receiving immunosuppressants (table 2). The lowest seroconversion rate was identified in patients receiving methotrexate (seven [47%; 95% CI 21 to 73] of 15 patients seroconverted). Seroconversion in patients receiving TNF inhibitors (19 [79%; 95% CI 58 to 93] of 24) and IL-23 inhibitors (19 [83%; 61 to 95] of 23) was also lower than in controls, but to a lesser extent than in patients on methotrexate. In summary, methotrexate was associated with lower seroconversion rates than either healthy controls (β=–0·76 [95% CI −1·05 to −0·48]; p=0·0001) or targeted biological therapy (β=–0·52 [–0·77 to −0·26]; p=0·0001).

**Table 2:** Immunogenicity of the first dose of the COVID-19 vaccine BNT162b2

		**Anti-SARS-CoV-2 IgG (serological) vaccine response**		**T-cell vaccine response**	
		Number of responders	Proportion of responders (95% CI)	Number of responders	Proportion of responders (95% CI)
Healthy controls		17/17	100% (80–100)	11/16	69% (41–89)
Patients on immunosuppressants		60/77	78% (67–87)	65/77	84% (74–92)
	Patients on methotrexate	7/15	47% (21–73)	14/15	93% (68–100)
	Patients on TNF inhibitors	19/24	79% (58–93)	19/24	79% (58–93)
	Patients on IL-17 inhibitors	15/15	100% (78–100)	14/15	93% (68–100)
	Patients on IL-23 inhibitors	19/23	83% (61–95)	18/23	78% (56–93)

A threshold EC_50_ value of 25 was used for anti-SARS-CoV-2 IgG titres, at which serological responses were classified as positive. A threshold value of 30 cytokine-secreting cells per million peripheral blood mononuclear cells was established for total T-cell responses (interferon-γ, IL-2, or IL-21), at which the T-cell response was classified as positive. EC_50_=half maximal effective concentration. Humoral and cellular data were missing for two patients on methotrexate, three patients on TNF inhibitors, and two patients on IL-23 inhibitors. No cellular data were available for one healthy control. IL=interleukin. TNF=tumour necrosis factor.

**Figure 2:**
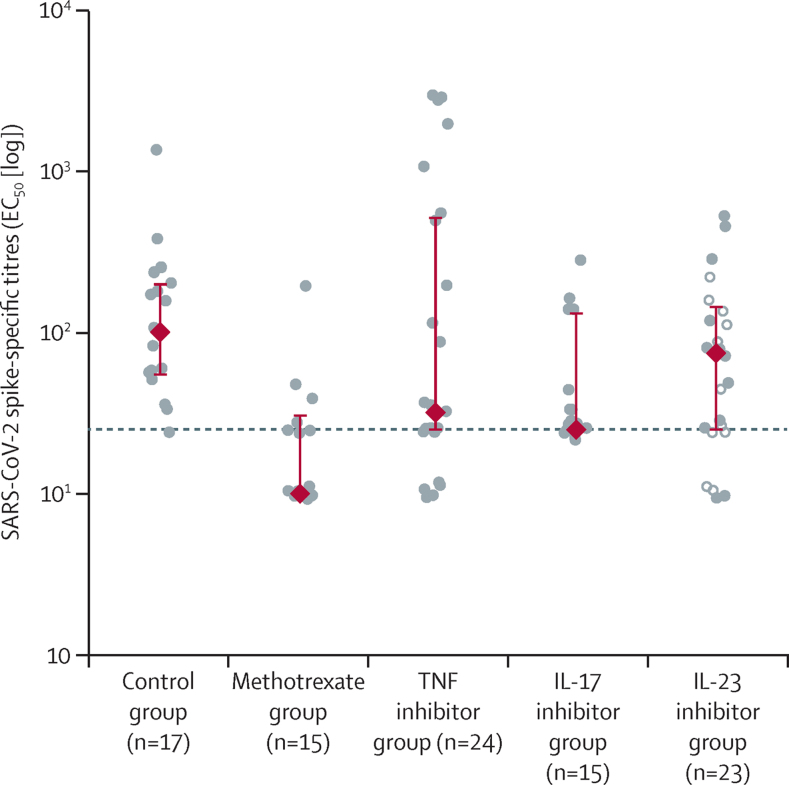
Serological immune responses to COVID-19 vaccine BNT162b2 Spike-specific IgG titres (EC_50_) in plasma samples on day 28 after vaccination in healthy controls and patients with psoriasis receiving methotrexate or targeted biological monotherapy. The circles represent individual values. The red diamonds and range lines indicate the median and IQR. In the IL-23 inhibitor group, filled circles represent participants receiving IL-23p19 inhibitors and hollow circles represent participants receiving an IL-12/23p40 inhibitor. The horizontal dashed line indicates the seroconversion threshold. EC_50_=half maximal effective concentration. IL=interleukin. TNF=tumour necrosis factor.

Of those who mounted a serological response, patients with psoriasis receiving immunosuppressants had lower median spike-specific IgG titres (half maximal effective concentration [EC_50_] 43 [IQR 25–162]) 28 days after the first dose of BNT162b2 than controls (101 [55–200]; figure 2; appendix p 9). At 28 days after vaccination, patients receiving methotrexate had significantly lower median anti-spike IgG titres (31 [IQR 25–48], p=0·015) than controls. Median spike-specific IgG titres were numerically, but not significantly, lower in patients receiving methotrexate than in those receiving targeted biological therapy (48 [IQR 25–174], p=0·26).

**Figure 3:**
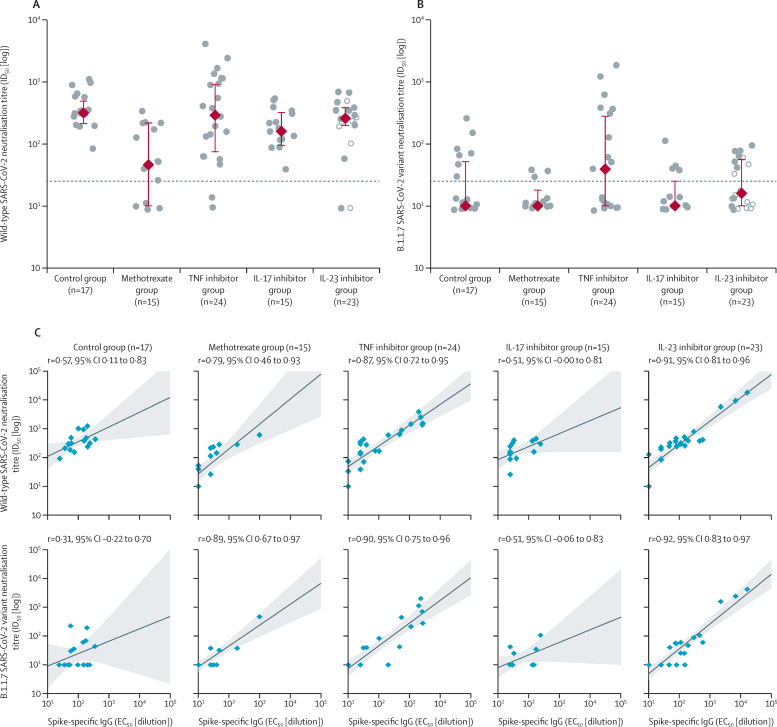
Functional humoral immunogenicity of the COVID-19 vaccine BNT162b2 (A) Neutralisation titres against wild-type SARS-CoV-2 on day 28 after the first dose of BNT162b2. (B) Neutralisation titres against B.1.1.7 SARS-CoV-2 variant on day 28 after the first dose of BNT162b2. The circles represent individual values. The red diamonds and range lines indicate the median and IQR. In the IL-23 inhibitor group, filled circles represent participants receiving IL-23p19 inhibitors and hollow circles represent participants receiving an IL-12/23p40 inhibitor. The horizontal dashed line indicates neutralisation activity threshold. (C) Correlation between spike-specific IgG and neutralisation titres against wild-type or the B.1.1.7 variant. Blue diamonds show individual patients, shading indicates the 95% CI. EC_50_=half maximal effective concentration. ID_50_=50% inhibitory dilution. IL=interleukin. TNF=tumour necrosis factor.

To further interrogate the observed differences in serological responses to BNT162b2 between individuals receiving methotrexate versus targeted immunosuppression, we next assessed the functional effect of seroconversion. Of those who had evidence of neutralisation activity, individuals receiving TNF inhibitors, IL-17 inhibitors, and IL-23 inhibitors had neutralisation titres (50% inhibitory dilution [ID_50_]) against wild-type SARS-CoV-2 that were similar to controls (figure 3A). By contrast, patients receiving methotrexate had lower median neutralisation titres (ID_50_ 129 [IQR 40–236]) than healthy controls (317 [213–487], p=0·0032) or patients receiving targeted biological therapy (269 [141–418], p=0·011).

Neutralisation activity against the B.1.1.7 variant was low across all study participants after the first dose of BNT162b2 (figure 3B), and there were no differences between controls and patients receiving immunosuppressants (median ID_50_ 61 [IQR 36–195] *vs* 54 [38–108], p=0·92).

Although seroconversion was determined by the presence of IgG binding to spike (without assessment of neutralising capacity), plasma neutralisation can be mediated by IgM, IgA, and IgG. Nonetheless, anti-spike IgG titres positively correlated with neutralisation titres for both the wild-type and B.1.1.7 strains in each study group (figure 3C). Neutralisation titres for wild-type and B.1.1.7 strains were also correlated (appendix p 10).

The frequency of T cells with a Th1 cytokine profile (characterised by IL-2 and interferon-γ) is widely used to assess vaccine-induced cellular immunity, including memory T-cell responses,^17^ and T follicular helper (Tfh)-derived IL-21 is crucial for memory B-cell responses.^18^ Receiver operator characteristic curve analysis established threshold values to determine a positive response for the total T-cell response (Th1 or Tfh; a combination of interferon-γ, IL-2, and IL-21) and for each individual cytokine (appendix p 11). All study groups showed a wide range of cytokine responses to peptides derived from commonly encountered viruses and fungal antigens, with no major differences in the magnitude or polarisation of response in any group (appendix p 12).

**Figure 4:**
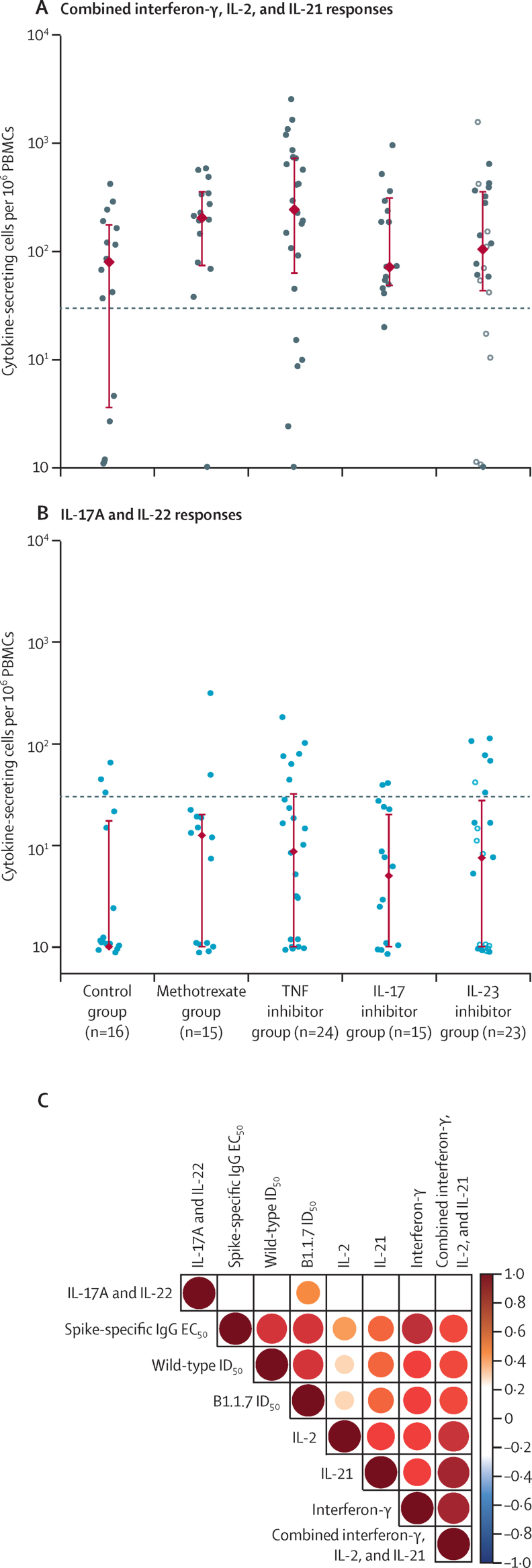
Cellular immunogenicity of the COVID-19 vaccine BNT162b2 (A) Total T-cell response, as determined by combined interferon-γ, IL-2, and IL-21 responses to stimulation with peptides from total spike peptide pools, reported as number of cytokine-secreting cells per 10^6^ cells in PBMC samples on day 28 after the first dose of BNT162b2. The horizontal line indicates the T-cell response threshold. (B) IL-17A and IL-22 responses to stimulation with peptides from total spike peptide pools, reported as number of cytokine-secreting cells per 10^6^ cells in PBMC samples on day 28 after the first dose of COVID-19 vaccine BNT162b2. The horizontal line indicates the T helper 17 response threshold. The circles represent individual values. The red diamonds and range lines indicate the median and IQR. In the IL-23 inhibitor group, filled circles represent participants receiving IL-23p19 inhibitors and hollow circles represent participants receiving an IL-12/23p40 inhibitor. (C) Spearman correlation between T-cell responses (cytokine-secreting cells per 10^6^ PBMCs) and humoral immune responses as determined by ELISA and neutralisation assays. The colour scale indicates the Spearman *R* values; all p values are less than 0·01. EC_50_=half maximal effective concentration. ID_50_=50% inhibitory dilution. IL=interleukin. PBMCs=peripheral blood mononuclear cells. TNF=tumour necrosis factor.

All study groups showed positive spike-specific T-cell responses 28 days after the first dose of BNT162b2, as evidenced by production of interferon-γ, IL-2, or IL-21 (figure 4A; appendix p 13). T-cell response rates were similar in patients receiving methotrexate and targeted biologics and in controls (table 2). In regression analyses, methotrexate was not associated with lower cellular immunogenicity compared with healthy controls (β=0·65 [95% CI 0·07 to 1·24], p=0·029) or targeted biological therapy (β=0·12 [–0·28 to 0·53], p=0·53). The magnitude of T-cell responses was also similar between all study groups, both with respect to the combined interferon-γ, IL-2, and IL-21 response (figure 4A) and for individual cytokines (appendix p 14).

T-cell reactivities were also evident in serological non-responders (individuals without evidence of seroconversion) who were receiving methotrexate (seven of eight), TNF inhibitors (three of five), and IL-23 inhibitors (one of four). Thus, T-cell responses were successfully induced following vaccination in patients receiving either methotrexate or targeted biologics, with similar levels of response across treatment groups, independently of detectable humoral immunogenicity.

Given the central role of Th17 immunity in the pathogenesis of psoriasis, the therapeutic efficacy of targeted IL-17 blockade,^10^ and the association between Th17 cell activation and severe COVID-19,^15^, ^16^ we next analysed IL-17A and IL-22 cytokine responses to total spike peptide pools across the study groups. IL-17A and IL-22 responses were low across all four immunosuppressant groups, and similar to responses seen in controls (figure 4B). Collectively, interferon-γ, IL-2, and IL-21 T cell responses (in particular, interferon-γ) broadly correlated with serological and functional humoral immune responses (figure 4C). By contrast, no correlation was observed between Th17 type responses and humoral immunity. However, there was variation in the strength of correlation across cytokines and drug categories (appendix p 15).

Finally, we assessed humoral and cellular immune responses of four individuals receiving immunosuppression therapy (one on methotrexate and three on IL-23 inhibitors) who received the first dose of BNT162b2 but were excluded from the main study due to previous confirmed natural SARS-CoV-2 infection. On analysis of their humoral immunity, there was evidence of robust serological and neutralising responses against both wild-type SARS-CoV-2 and the B.1.1.7 variant 28 days after vaccination (appendix p 16). Similarly, robust cellular immune responses were evident at day 28 (appendix p 16). These humoral and cellular immune responses exceeded that of our infection-naive study participants receiving the same immunosuppression therapy (and controls).

## Discussion

This study characterises the humoral and cellular immune response to the first dose of BNT162b2 vaccine in individuals receiving monotherapy with methotrexate, TNF inhibitors, IL-17 inhibitors, or IL-23 inhibitors. Our homogeneous cohort of individuals receiving immunosuppressant monotherapy for psoriasis, without concurrent corticosteroids, has enabled the interrogation of drug-specific (rather than disease-specific) effects on vaccine immunogenicity. We show that seroconversion and neutralising capacity against the wild-type SARS-CoV-2 strain is lower in patients receiving methotrexate than in those receiving targeted biological therapy or healthy controls. Neutralising titres against the B.1.1.7 variant were low for all groups. Notably, however, cellular immunity following BNT162b2, including Th1 and Tfh responses, was shown across all classes of immunosuppression and was similar to that of controls and also similar to levels observed in other control cohorts.^11^, ^14^

Although our findings on vaccine immunogenicity in the context of targeted biologics are encouraging, the results from patients treated with methotrexate highlight a disparity between humoral and cellular immunogenicity. The clinical significance of this finding warrants further research. Our study builds on recent research, which has primarily assessed only serological responses to COVID-19 vaccines in immunosuppressed patients, without considering neutralising antibody responses (the gold standard measure of humoral immunogenicity) or cellular immunity.^19^, ^20^, ^21^, ^22^ Consistent with our data indicating lower levels of spike-specific IgG in patients receiving TNF inhibitors versus controls, a study of 1293 patients with inflammatory bowel disease showed lower antibody titres and seroconversion rates following a single dose of vaccine in those receiving TNF blockade (infliximab) than in a reference cohort of patients receiving vedolizumab (gut-selective integrin inhibitor).^19^ Multivariable models highlighted an association of age of 60 years or older and co-therapy (azathioprine, mercaptopurine, or methotrexate) with lower SARS-CoV-2 antibody titres; however, neutralising titres or cellular immunity (which were similar in our TNF inhibitor cohort versus controls) were not explored. Serological responses to a single dose of vaccine were also lower in recipients of solid organ transplants receiving anti-metabolite immunosuppression (mycophenolate mofetil, mycophenolic acid, or azathioprine) than in those not receiving such immunosuppression.^22^ Finally, both cellular and humoral immunogenicity of a single dose of vaccine was low in immunocompromised individuals with solid and haematological cancers.^14^ This finding contrasts with our data showing intact cellular responses in immunosuppressed individuals with immune-mediated inflammatory diseases and might be attributable to the effect of the underlying malignancy and type or burden of therapeutic immunosuppression.^14^ In our dataset, the first dose of BNT162b2 induced favourable T-cell responses (interferon-γ, IL-2, and IL-21) to total spike peptide pools in the majority of controls and patients receiving immunosuppressants, with no attenuation in cellular immunity found in those receiving methotrexate or targeted biological monotherapy compared with controls. Furthermore, T cells from patients receiving methotrexate or targeted biologics who were serological non-responders demonstrated T-cell reactivities following the vaccine, thus highlighting a disparity between humoral and cellular responses.

The true in-vitro correlates of immune protection after vaccination, and the relative importance of humoral and cellular immunity, are unknown. So far research on responses to the COVID-19 vaccine has overwhelmingly focused on serological responses, and there is a wide variation in laboratory methodology used (limiting comparability of assays) and no indication of the level of antibody required for protection (ie, clinical effectiveness). Furthermore, the importance of cellular immune responses in preventing SARS-CoV-2 transmission in the context of diminished humoral immunity is still unclear. Vaccine-induced cell-mediated immunity might be a better surrogate than humoral immunity for protection against respiratory viruses such as influenza in patients with compromised immune responses.^23^, ^24^ Cellular immunity has been shown to be crucial for clearance of SARS-CoV-2 and recovery from COVID-19.^25^, ^26^ SARS-CoV-2-specific CD4 and CD8 T-cell responses were observed in patients with COVID-19,^27^ and memory B cells and Tfh cells were identified following recovery. T cells recognising peptides derived from the spike glycoprotein, nucleoprotein, and matrix of SARS-CoV-2 were found in the convalescent phase, and low numbers of T cells, T-cell exhaustion, and increased levels of pro-inflammatory cytokines are hallmarks of severe COVID-19. Thus, cellular responses after vaccination might affect overall vaccine efficacy against SARS-CoV-2. Given the known effect of methotrexate and biological therapies on T-cell proliferation, activation, and cytokine production, our data on cell-mediated responses after vaccination are encouraging.

Our findings support existing data showing that methotrexate attenuates humoral immunity; methotrexate is used therapeutically to prevent anti-drug antibody development and it decreases vaccine serological responses to seasonal influenza (particularly novel strains) and pneumococcus.^8^, ^28^ Trial data in rheumatoid arthritis indicate that temporary methotrexate discontinuation for 2 weeks after influenza vaccination might increase seroprotection rates compared with continuing methotrexate.^29^ However, only humoral immunogenicity was assessed, and the clinical significance of higher antibody responses on the incidence of influenza infections is unknown. Registry data suggest that methotrexate or biological monotherapy for immune-mediated inflammatory diseases is not associated with adverse COVID-19 outcomes,^30^, ^31^ so it is unclear whether the reduced humoral responses to BNT162b2 in the context of methotrexate translate to an increased SARS-CoV-2 infection risk.

These data also highlight variation in vaccine immunogenicity against different variants of SARS-CoV-2. Novel variants harbouring mutations in the spike glycoprotein might evade vaccine-induced protective immunity, and the highly transmissible B.1.1.7 variant harbouring nine amino acid changes in the spike protein has been shown to have increased resistance to neutralisation by antibodies generated following vaccination or infection.^32^, ^33^, ^34^ While assessing responses following the second vaccine dose is vital, we identified low neutralisation titres against B.1.1.7 after vaccination in both controls and patients with psoriasis receiving immunosuppressants, which underlines the need for ongoing risk mitigating measures following the first dose of vaccine. Emerging evidence indicates that neutralising antibody titres against B.1.1.7 generated by vaccination might not correlate with clinical vaccine effectiveness against symptomatic COVID-19,^35^ so the contribution of cellular immunity (T-cell and natural killer cell responses and antibody-dependent cellular cytotoxicity or phagocytosis) in vaccine-induced protection against new lineages of SARS-CoV-2 warrants investigation.

Our study addresses several limitations of existing datasets. We have interrogated the effect of different immunosuppressant treatment types on both humoral and cellular vaccine immune responses in a relatively young and homogeneous study cohort. Our study has limited confounding since participants had the same underlying immune-mediated inflammatory disease (psoriasis) with low to absent disease activity, and they were not receiving combination therapy with other immunosuppressants or glucocorticoids. The major classes of biologics used across immune-mediated inflammatory diseases were included, in addition to the widely prescribed first-line immunosuppressant methotrexate. Limitations of our study include a limited numbers of participants and absence of immunogenicity data following the second (booster) dose of vaccine. Although the number of participants was small, study groups were well matched with respect to baseline demographics and multiple components of the immune response are profiled, which will inform future larger-scale studies. Longer-term data are not presented since this interim analysis focused on characterising immune efficacy of the priming dose of the vaccine. Encouragingly, the robust humoral and cellular responses observed following the first dose of BNT162b2 in the four patients receiving immunosuppression with past SARS-CoV-2 infection indicates infection priming of immune responses. This is in keeping with observations in immunocompetent individuals.^11^ Important follow-up immunogenicity data following the (extended 12 week interval) booster dose of vaccine in our infection-naive participants receiving immunsuppression are anticipated.

While global mass COVID-19 vaccination programmes are underway, there remains concern over vaccine efficacy in immunosuppressed patients, including against novel SARS-CoV-2 variants that threaten immune escape. Measures of the immune response that correspond to risk of COVID-19 after vaccination are currently unknown and emerging research in immunosuppressed patients has focused on serological responses alone. We show that serological responses are not representative of the complex immune response to vaccines, and thus might have limited clinical utility when considered in isolation. Although our data indicating preserved cellular immunogenicity across biological classes and methotrexate, independent of neutralising activity, are reassuring, ongoing pharmacovigilance studies will determine whether this finding translates into clinical effectiveness of vaccines. The magnitude and quality of T-cell responses will also be affected by further vaccine doses, so analysing cellular dynamics following booster doses is important. Nonetheless, the observed attenuated functional humoral immunogenicity of the first dose of BNT162b2 in individuals receiving methotrexate and low neutralising activity against B.1.1.7 in all study participants indicate a reduced time interval between initial and booster vaccine doses might be favourable. These data underscore the need for ongoing stringent risk mitigating measures after a first dose of vaccine to reduce exposure to SARS-CoV-2.^36^

## Data sharing

The authors are happy to share data on request to the corresponding author.

## Declaration of interests

CHS reports grants from AbbVie, Sanofi, Novartis, and Pfizer, outside the submitted work. JBG reports personal fees from AbbVie, Sanofi, Novartis, Pfizer, Janssen, and UCB, and grants from Eli Lilly, outside the submitted work. SKM reports departmental income from AbbVie, Celgene, Eli Lilly, Janssen-Cilag, Novartis, Sanofi, and UCB, outside the submitted work. MAB reports departmental income from Clementia, Eli Lilly, Ipsen, Novartis, Pathios Therapeutics, Regeneron, and UCB, outside the submitted work. JNB reports grants and personal fees from AbbVie, Novartis, Lilly, and J&J, outside the submitted work. SN reports personal fees from Pfizer and Janssen, outside of the submitted work. APC reports grants from Bristol Myers Squibb and Janssen, and speaker bureau and consultancy fees from AbbVie, Bristol Myers Squibb, and UCB. TIMT reports grants from GlaxoSmithKline, Sanofi, and Imcyse, and consultancy fees from GlaxoSmithKline, Novartis, UCB, and Quell Therapeutics, outside the submitted work. All other authors declare no competing interests.
