# Supplementary material for: The effect of methotrexate and targeted immunosuppression on humoral and cellular immune responses to the COVID-19 vaccine BNT162b2: a cohort study
Source: Lancet Rheumatol. 2021 Jul 8;3(9):e627–37. doi: 10.1016/S2665-9913(21)00212-5 (PMC8266273; doi:10.1016/S2665-9913(21)00212-5)
Supplement: Supplementary appendix [file mmc1.pdf]

THE LANCET
Rheumatology

Supplementary appendix

This appendix formed part of the original submission and has been peer reviewed. We post it as supplied by the authors.

Supplement to: Mahil S K, Bechman K, Raharja A, et al. The effect of methotrexate and targeted immunosuppression on humoral and cellular immune responses to the COVID-19 vaccine BNT162b2: a cohort study. *Lancet Rheumatol* 2021; published online July 8. https://doi.org/10.1016/S2665-9913(21)00212-5.

Appendix

Section A: Supplementary Methods

Section B: Supplementary Tables

Section C: Supplementary Figures

Section A: Supplementary Methods

Humoral immunogenicity assays

Seroconversion was assessed using enzyme-linked immunosorbent assays (ELISA) as previously described[1]. Briefly, high-binding ELISA plates (Corning, 3690) were coated overnight at 4°C or for 2 hours at 37 °C with antigen (N protein or S glycoprotein at 3µg ml^{-1} in PBS). Wells were washed with PBS-T (PBS with 0.05% Tween-20) and blocked with 5% milk in PBS-T for 1 hour at room temperature. Serial dilutions of heat-inactivated plasma (starting at 1:25, 6-fold dilution) were added to wells containing S and plasma at 1:25 dilution was added to wells containing N. Plasma was incubated for 2 hours at room temperature. Control reagents included CR3009 (2 µg ml^{-1}) (N-specific monoclonal antibody), CR3022 (0.2 µg ml^{-1}) (S-specific monoclonal antibody), negative control plasma (1:25 dilution), positive control plasma (1:50) and blank wells. Wells were incubated with secondary antibody for 1 hour at room temperature. IgG was detected using goat-anti-human-Fc-AP (alkaline phosphatase) (1:1,000) (Jackson). AP substrate (Sigma) was added, and plates read in duplicate at 405 nm. EC50 values were calculated in GraphPad Prism. Where an EC50 was not reached at 1:25, a plasma was considered seropositive if the OD at 405nm was 4-fold above background and a value of 25 was assigned.

To assess the functional impact of seroconversion, we measured the capacity of participants' plasma to neutralise both the prototypic (wild-type) strain of SARS-CoV-2 and the highly transmissible B.1.1.7 variant which contains nine (protein sequence) mutations in Spike. Pseudotyped HIV particles incorporating the SARS-CoV-2 Wuhan-Hu-1 Spike (hereafter referred to as wild-type [WT]) or B.1.1.7 variant Spike were prepared as previously described[1]. Serial dilutions of heat inactivated plasma samples were prepared in DMEM complete media (10% fetal bovine serum (FBS) and 1% Pen/Strep (100 IU/mL penicillin and 100 ug/mL streptomycin) and incubated with WT or B.1.1.7 pseudotyped virus for 1 hour at 37 °C in 96-well plates. HeLa cells stably expressing the ACE2 receptor (provided by Dr James Voss, Scripps Research, La Jolla, CA) were then added (12,500 cells/50µL per well) and the plates left for 72 hours. Infection level was assessed in lysed cells with the Bright-Glo luciferase kit (Promega), using a Victor™ X3 multilabel reader (Perkin Elmer). The ID_{50} was calculated from duplicate measurements using GraphPad Prism.

Cellular immunogenicity assays

PBMCs were frozen at -80°C in CS10 CryoStor following isolation from blood samples, before transfer to liquid nitrogen storage. Cryopreserved PBMCs from vaccinated individuals were thawed and viability assessed by trypan blue exclusion following a resting period, shown by others to facilitate a more accurate enumeration of cell viability and and improved detection of antigen specific responses by cytokine ELISPOT. PBMC were stimulated with two separate SARS-CoV-2 spike protein peptide pools, one spanning the receptor binding domain containing S1 domain, and the other the S2 domain (PepMix SARS-CoV-2 spike pools 1+2 consisting of 158+157 15mer peptides spanning the S1 and S2 domain of the spike protein, JPT Peptide Technologies). These peptide pools are capable of stimulating both MHC-I and MHC-II restricted T cells with no particular HLA bias. PBMCs were also stimulated with CEF (CMV, EBV, influenza virus HLA class I epitopes) + CEFT (CMV, EBV, influenza

virus, tetanus toxoid HLA class II epitopes) + Candida albicans antigen (Greer Laboratories) as a positive control or DMSO, as a negative control.

All peptides were used at a final concentration of 0.25µg/ml/peptide and Candida albicans antigen at 0.5µg/ml. IFNγ, IL-2, IL-17A/IL-22 responses were detected by a direct FluoroSpot assay and IL-21 was detected by a direct ELISPOT assay (MabTech, Sweden) according to the manufacturer's instructions. Briefly, $2x10^5$ PBMCs were transferred to wells of pre-coated FluoroSpot and ELISPOT plates and stimulated in duplicate for 46-48h with the stimuli described above. Cells were removed by washing, and antibody-bound cytokines identified using a cocktail of anti-IFN-γ-BAM, anti-IL-2-WASP and anti-IL-17A/IL-22-biotinylated detection antibodies, followed by three fluorescent conjugates: anti-BAM (IFN-γ), anti-WASP (IL-17A) and Streptavidin-550 (IL-17A and IL-22) according to manufacturer's instructions. IL-21 was detected by a biotin-bound detection antibody followed by streptavidin-ALP. The substrate solution BCIP/NBT was added, and plates developed until well-defined spots emerged. Plates were scanned using AID *i*Spot Spectrum reader and analysed using AID EliSpot 8.0' software (AID Autoimmun Diagnostika). Data were analysed using a quality controlled standard operating procedure and values expressed as cytokine secreting cells/10^6 PBMC following subtraction of values from negative control wells. Total response to SARS-CoV-2 spike protein was determined by summing responses to spike pools 1+2 to derive total spike specific T cell frequency. For every batch, a replicate cryopreserved PBMC sample from a single blood draw from a vaccinated control individual was included as a positive control. A threshold value of 30 cytokine secreting cells per million PBMCs was established, at which the total T cell response was classified as positive (Appendix Figure 4A).

References

1. Monin, L. *et al.* Safety and immunogenicity of one versus two doses of the COVID-19 vaccine BNT162b2 for patients with cancer: interim analysis of a prospective observational study. *Lancet Oncol.* (2021) doi:10.1016/S1470-2045(21)00213-8.

Section B: Supplementary Tables

Table 1: Baseline characteristics of study participants, disaggregated by sex.

	HV		MTX		TNF		IL17		IL23		Total		P value
Number in each group	F (n=8)	M (n=9)	F (n=6)	M (n=11)	F (n= 14)	M (n=13)	F (n=7)	M (n=8)	F (n=10)	M (n=15)	F (n=45)	M (n=56)	0.84†
Age (year)†	26.0 (23.5-43.5)	37.0 (29.0-46.0)	52.0 (46.0-56.0)	44.0 (39.0-57.0)	36.0 (28.0-52.0)	41.0 (28.0-51.0)	39.0 (37.0-48.0)	46.5 (40.5-51.0)	41.0 (28.0-50.0)	52.0 (33.0-60.0)	39.0 (28.0-51.0)	45.0 (33.0-53.0)	0.053*
BMI (mg/m2) †	23.5 (21.5-30.5)	23.0 (21.4-29.2)	29.4 (27.1-33.3)	26.5 (26.3-28.1)	28.9 (24.8-32.1)	32.0 (28.7-40.1)	26.3 (24.1-27.0)	30.1 (26.6-32.8)	29.0 (27.0-32.3)	28.4 (26.5-34.0)	27.9 (24.3-31.6)	28.2 (26.3-33.3)	0.032*
Ethnicity, n (%)													
White	7 (88%)	7 (78%)	6 (100%)	7 (64%)	13 (93%)	11 (85%)	6 (86%)	7 (88%)	9 (90%)	12 (80%)	41 (91%)	44 (79%)	0.72*
Black	0	0	0	1 (9%)	0	0	0	0	0	0	0	1 (2%)	
South Asian	1 (13)	2 (22.2%)	0	3 (27%)	1 (7%)	2 (15%)	1 (14%)	1 (13)	1 (10%)	2 (13%)	4 (8.9%)	10 (18%)	
Mixed	0	0	0	0	0	0	0	0	0	1 (7%)	0	1 (2%)	
Disease severity measure (psoriasis area severity index) †	-	-	2.4 (1.2-3.0)	2.2 (1.3-4.3)	0.9 (0.0-1.5)	1.5 (0.8-2.5)	0.0 (0.0-0.3)	1.6 (0.9-5.0)	1.8 (0.0-2.8)	1.2 (0.0-2.8)	0.9 (0.0-2.0)	1.8 (0.8-3.5)	0.11†
Concomitant psoriatic arthritis, n (%)	-	-	2 (33%)	0	1 (7%)	4 (31%)	4 (57%)	4 (50%)	2 (20%)	3 (20%)	9 (20%)	11 (19%)	0.027*

All values are given as numbers (%) unless otherwise specified. † Median [IQR]. ** Total percentage of those with psoriasis.

Statistical imbalance of the baseline characteristics across the treatment groups presented by either kwallis or χ^2

HV, healthy volunteers; MTX, methotrexate; TNFi, TNF inhibitors; IL17i, IL-17 inhibitors; IL23i, IL-23 inhibitors.

Table 2: Adverse events, disaggregated by sex.

	HV		MTX		TNF		IL17		IL23		Total	
Number in each group	F (n=8)	M (n=9)	F (n=6)	M (n=11)	F (n= 14)	M (n=13)	F (n=7)	M (n=8)	F (n=10)	M (n=15)	F (n=45)	M (n=56)
Any Systemic Event	3 (38%)	4 (44%)	2 (33%)	3 (27%)	8 (62%)	6 (55%)	4 (57%)	3 (36%)	6 (60%)	4 (27%)	23 (53%)	20 (38%)
Chills	0 (0%)	1 (11%)	0 (0%)	0 (0%)	0 (0%)	0 (0%)	1 (14%)	0 (0%)	2 (20%)	0 (0%)	3 (7%)	1 (2%)
Fatigue	1 (13%)	4 (44%)	2 (33%)	1 (9%)	3 (23%)	3 (27%)	2 (27%)	2 (25%)	3 (30%)	3 (21%)	11 (25%)	13 (25%)
Fever	0 (0%)	0 (0%)	0 (0%)	0 (0%)	0 (0%)	0 (0%)	0 (0%)	0 (0%)	0 (0%)	0 (0%)	0 (0%)	1 (2%)
Headache	2 (25%)	1 (11%)	1 (17%)	3 (27%)	4 (31%)	3 (27%)	2 (27%)	1 (13%)	5 (50%)	1 (7%)	14 (32%)	9 (17%)
Malaise	1 (13%)	2 (22%)	0 (0%)	1 (9%)	2 (15%)	2 (18%)	1 (14%)	2 (25%)	3 (30%)	1 (7%)	7 (16%)	8 (15%)
Nausea	0 (0%)	0 (0%)	0 (0%)	0 (0%)	0 (0%)	1 (9%)	2 (27%)	2 (25%)	2 (20%)	0 (0%)	0 (0%)	3 (6%)
Any local Event	7 (88%)	8 (89%)	6 (100%)	6 (55%)	9 (69%)	8 (73%)	7 (100%)	6 (75%)	8 (80%)	10 (71%)	37 (84%)	38 (72%)
Induration	0 (0%)	0 (0%)	0 (0%)	0 (0%)	0 (0%)	0 (0%)	0 (0%)	0 (0%)	0 (0%)	1 (7%)	0 (0%)	1 (2%)
Itch	0 (0%)	0 (0%)	1 (17%)	0 (0%)	0 (0%)	0 (0%)	0 (0%)	0 (0%)	0 (0%)	0 (0%)	2 (5%)	0 (0%)
Pain	7 (88%)	7 (78%)	5 (83%)	6 (55%)	8 (62%)	8 (73%)	7 (100%)	5 (63%)	7 (70%)	9 (64%)	34 (77%)	35 (66%)
Redness	0 (0%)	0 (0%)	0 (0%)	0 (0%)	1 (8%)	0 (0%)	0 (0%)	0 (0%)	1 (10%)	0 (0%)	0 (0%)	0 (0%)
Swelling	0 (0%)	0 (0%)	0 (0%)	0 (0%)	0 (0%)	0 (0%)	0 (0%)	0 (0%)	1 (10%)	0 (0%)	0 (0%)	0 (0%)
Tender	5 (63%)	7 (78%)	6 (100%)	4 (36%)	4 (31%)	6 (55%)	2 (27%)	3 (36%)	7 (70%)	4 (27%)	24 (55%)	24 (45%)
Warmth	0 (0%)	0 (0%)	0 (0%)	0 (0%)	0 (0%)	0 (0%)	0 (0%)	0 (0%)	0 (0%)	0 (0%)	0 (0%)	0 (0%)

Section C: Supplementary Figures

Appendix Figure 1: Local and systemic adverse events reported within 28 days after the COVID-19 vaccine BNT162b2.

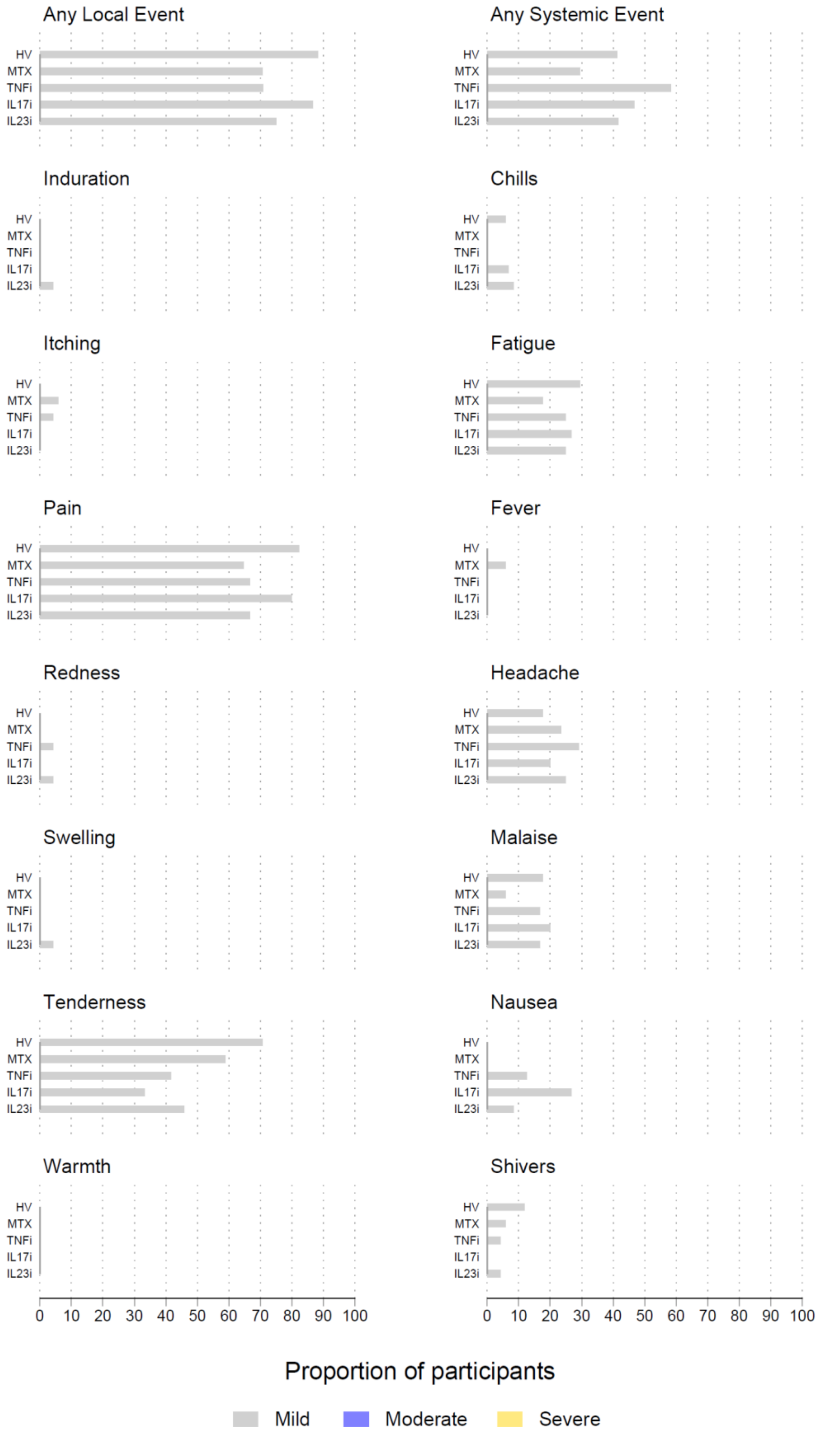

Percentage of participants in each study group reporting local and systemic adverse events following the first dose of BNT162b2 vaccine. Symptoms were assessed according to the following scale: mild (no/minimal interference with activity and no/minimal medical intervention required), moderate (limitation in activity and medical intervention required), and severe (marked limitation in activity and medical intervention required). HV, healthy volunteers; MTX, methotrexate; TNFi, TNF inhibitors; IL17i, IL-17 inhibitors; IL23i, IL-23 inhibitors.

Appendix Figure 2: Serological data on study participants at day 0 and 28 following COVID-19 vaccine BNT162b2.

Figure 2A. IgG titres to Nucleoprotein (N) at Day 0 and Day 28 following the first dose of COVID-19 vaccine BNT162b2.

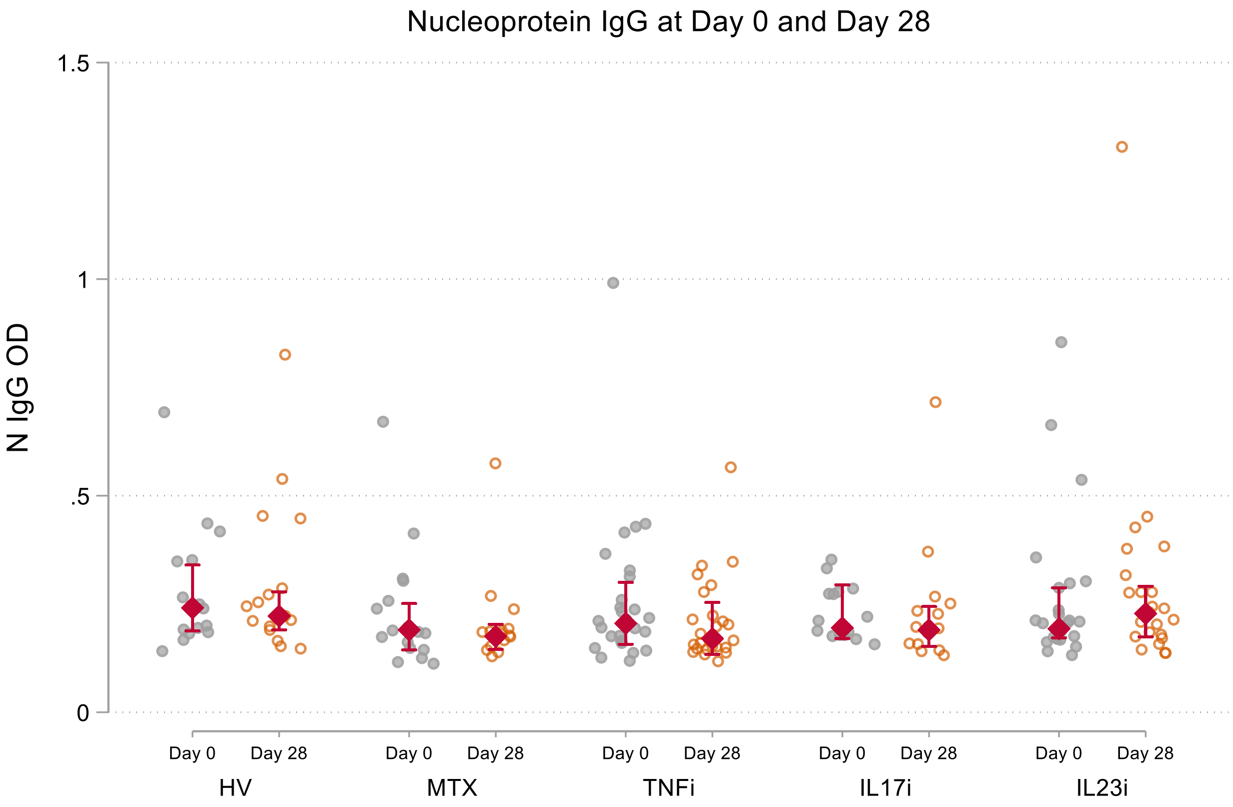

The circles represent individual values. The red diamonds and range lines indicate the median and interquartile range (IQR). HV, healthy volunteers; MTX, methotrexate; TNFi, TNF inhibitors; IL17i, IL-17 inhibitors; IL23i, IL-23 inhibitors. Absorbance (405nm).

Figure 2B. Spike-specific IgG at Day 0 and Day 28 following the first dose of COVID-19 vaccine BNT162b2

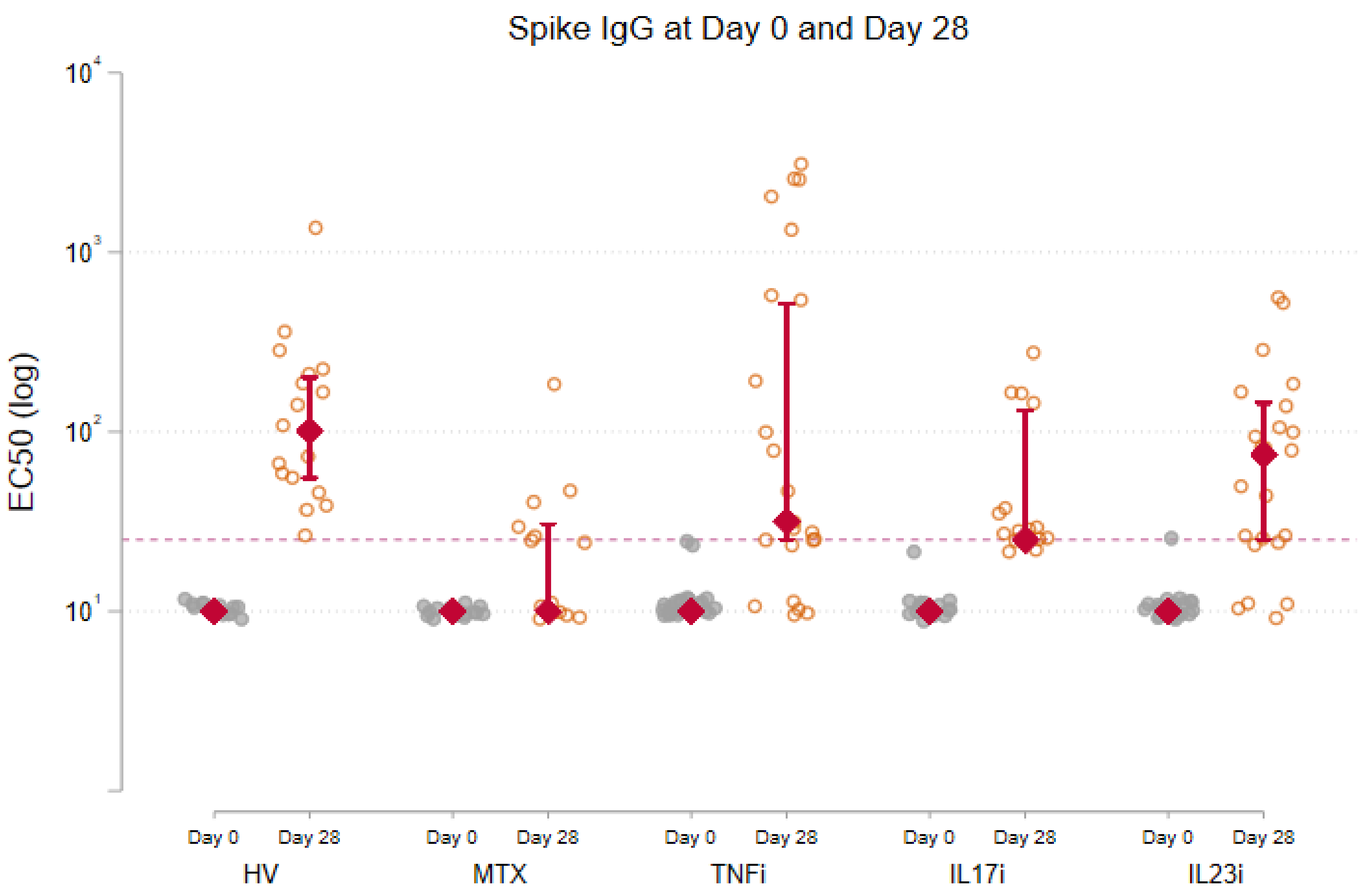

The circles represent individual values. The red diamonds and range lines indicate the median and interquartile range (IQR). HV, healthy volunteers; MTX, methotrexate; TNFi, TNF inhibitors; IL17i, IL-17 inhibitors; IL23i, IL-23 inhibitors.

Appendix Figure 3: Correlation between neutralization titres against WT and B.1.1.7 SARS-CoV-2 variants

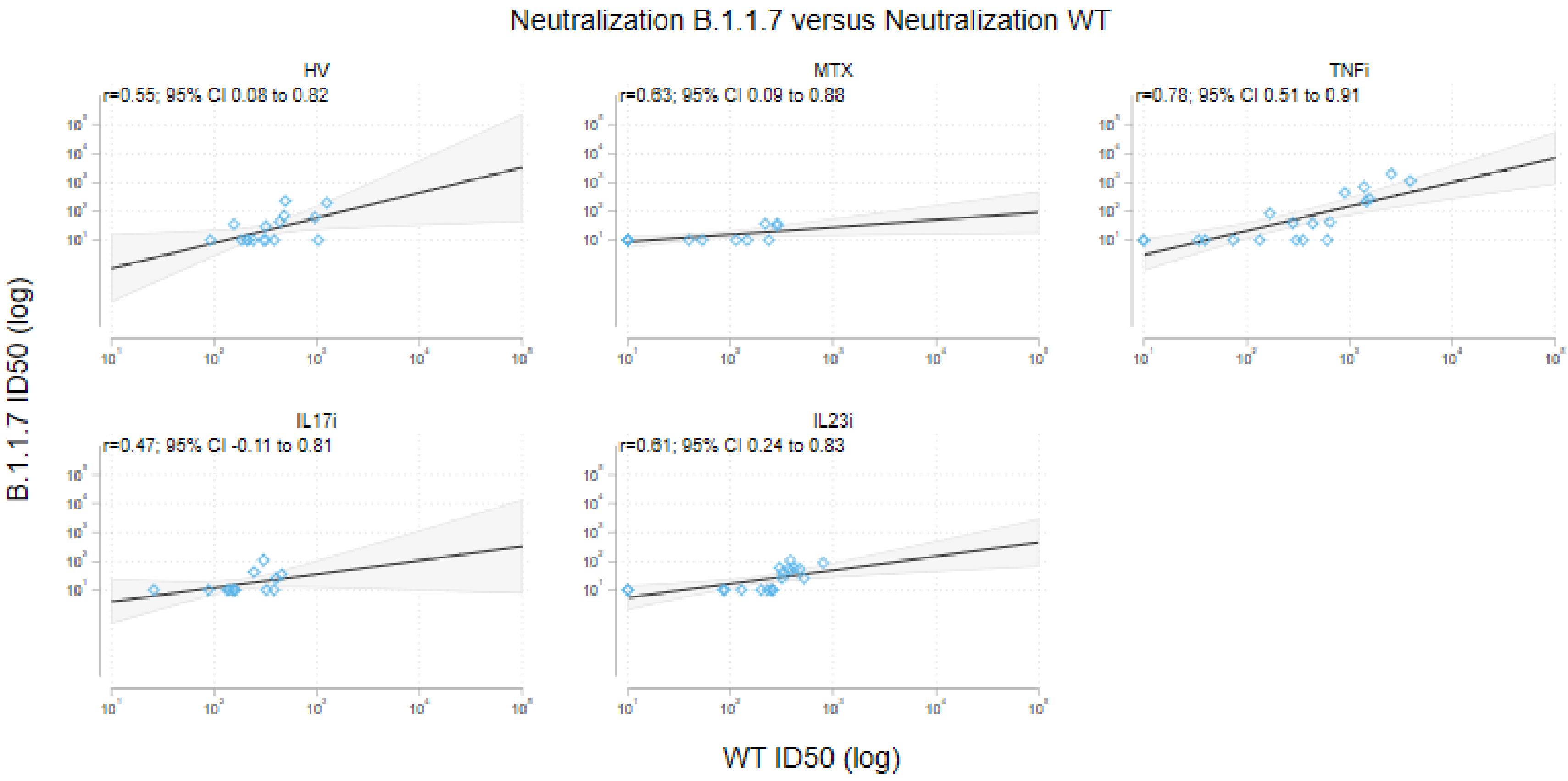

The diamonds represent individual values. N: total number individuals; r: rho by Pearson's correlation; 95 CI: 95% confidence interval. HV, healthy volunteers; MTX, methotrexate; TNFi, TNF inhibitors; IL17i, IL-17 inhibitors; IL23i, IL-23 inhibitors

Appendix Figure 4: QC and T cell responses to COVID-19 vaccine BNT162b2.

Figure 4A. Determining a cut-off value for assigning positive and negative cellular responses.

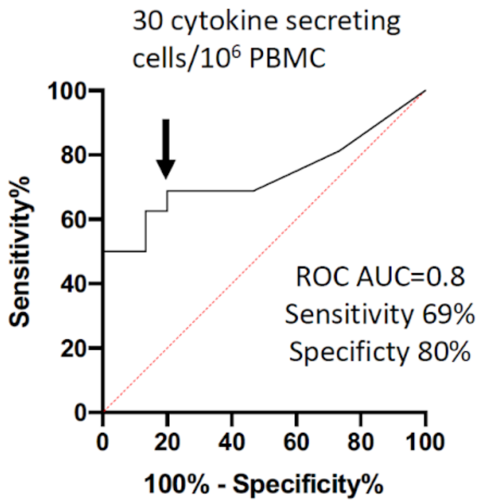

Graph represents a ROC plot showing assay diagnostic sensitivity against specificity (one minus proportion of false positives) following detection of summed IFN-, IL-2 and IL21 responses to Spike peptide pools in healthy control subjects before and following one dose of BNT162b2. By convention, we selected the cut-off value that provides an operating position nearest to that of the "perfect test" (i.e., closest approximation to 100% sensitivity and 100% specificity), which was ≥30 cytokine secreting cells/10^6 PBMC

Figure 4B. Positive control T cell responses to peptides derived from commonly encountered viruses and fungal antigens

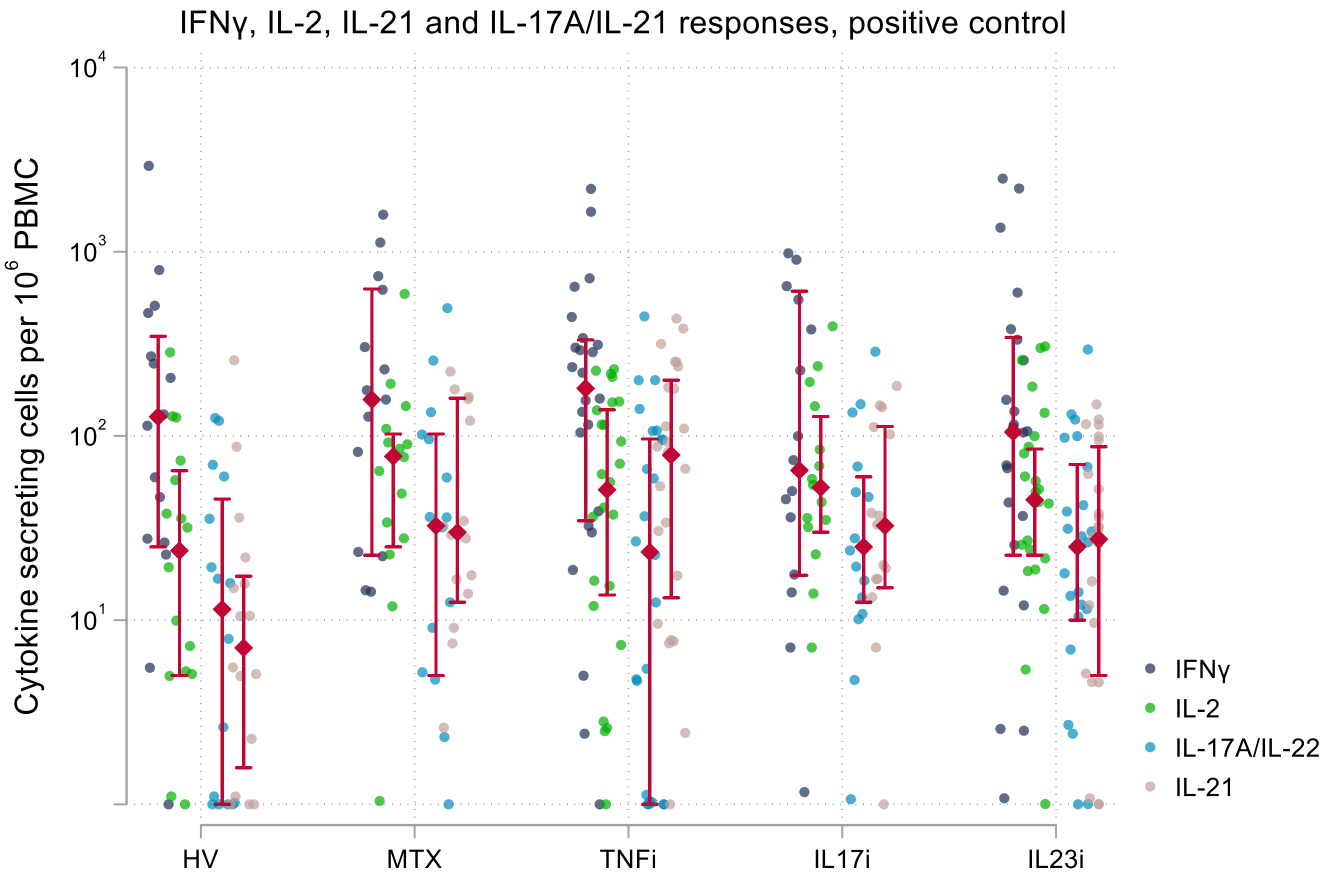

The positive control stimulus was CEF (CMV, EBV, influenza virus HLA class I epitopes) + CEFT (CMV, EBV, influenza virus, tetanus toxoid HLA class II epitopes) + Candida albicans antigen (Greer Laboratories). HV, healthy volunteers; MTX, methotrexate; TNFi, TNF inhibitors; IL17i, IL-17 inhibitors; IL23i, IL-23 inhibitors. The circles represent individual values. The red diamonds and range lines indicate the median and interquartile range (IQR).

Figure 4C. T cell response to negative control (DMSO), Spike pool 1 (S1) and Spike pool 2 (S2) as determined by IFNγ and/or IL-2 and/or IL-21 responses reported as number of cytokine secreting cells per 10^6 cells in PBMC.

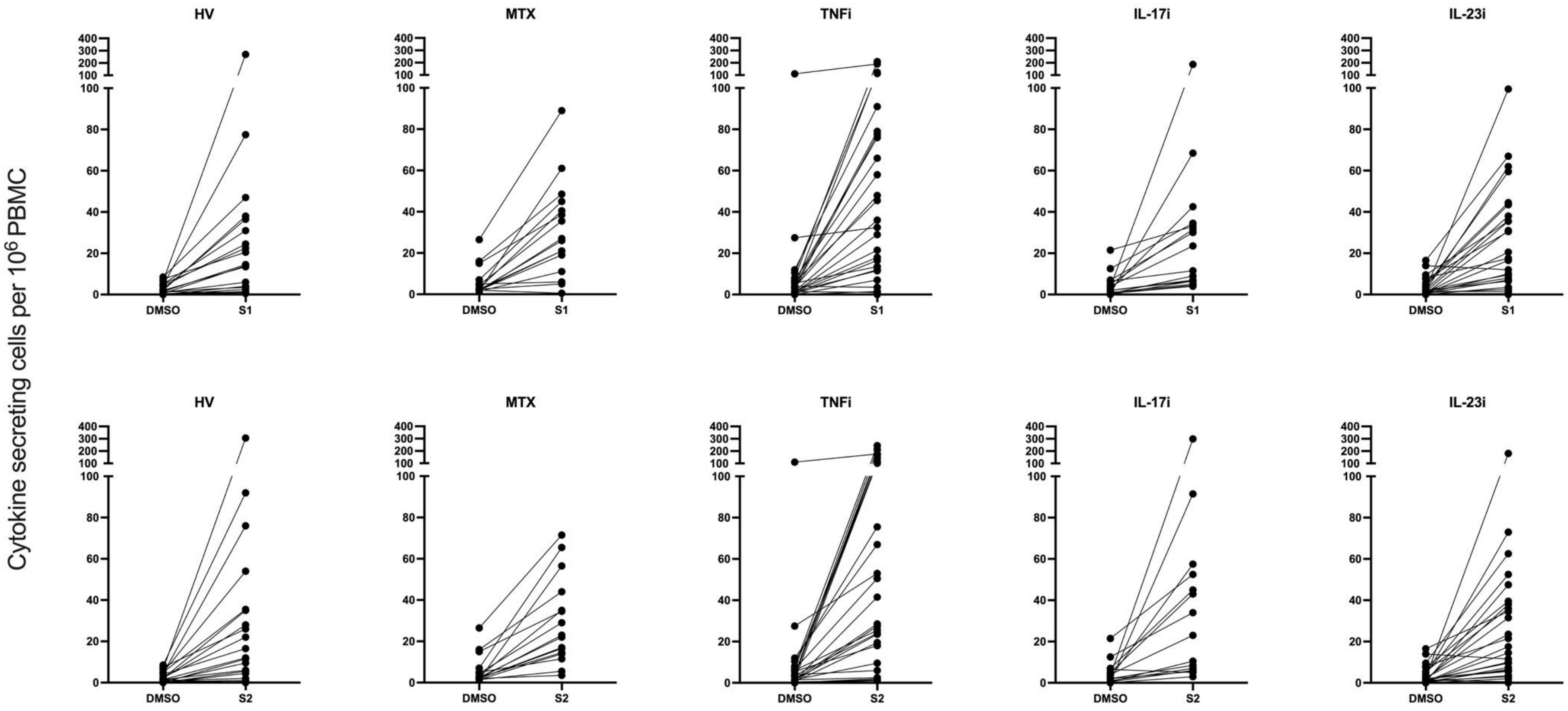

The circles represent individual values, lines join responses from the same individual

Figure 4D. Individual cytokine responses to the first dose of COVID-19 vaccine BNT162b2.

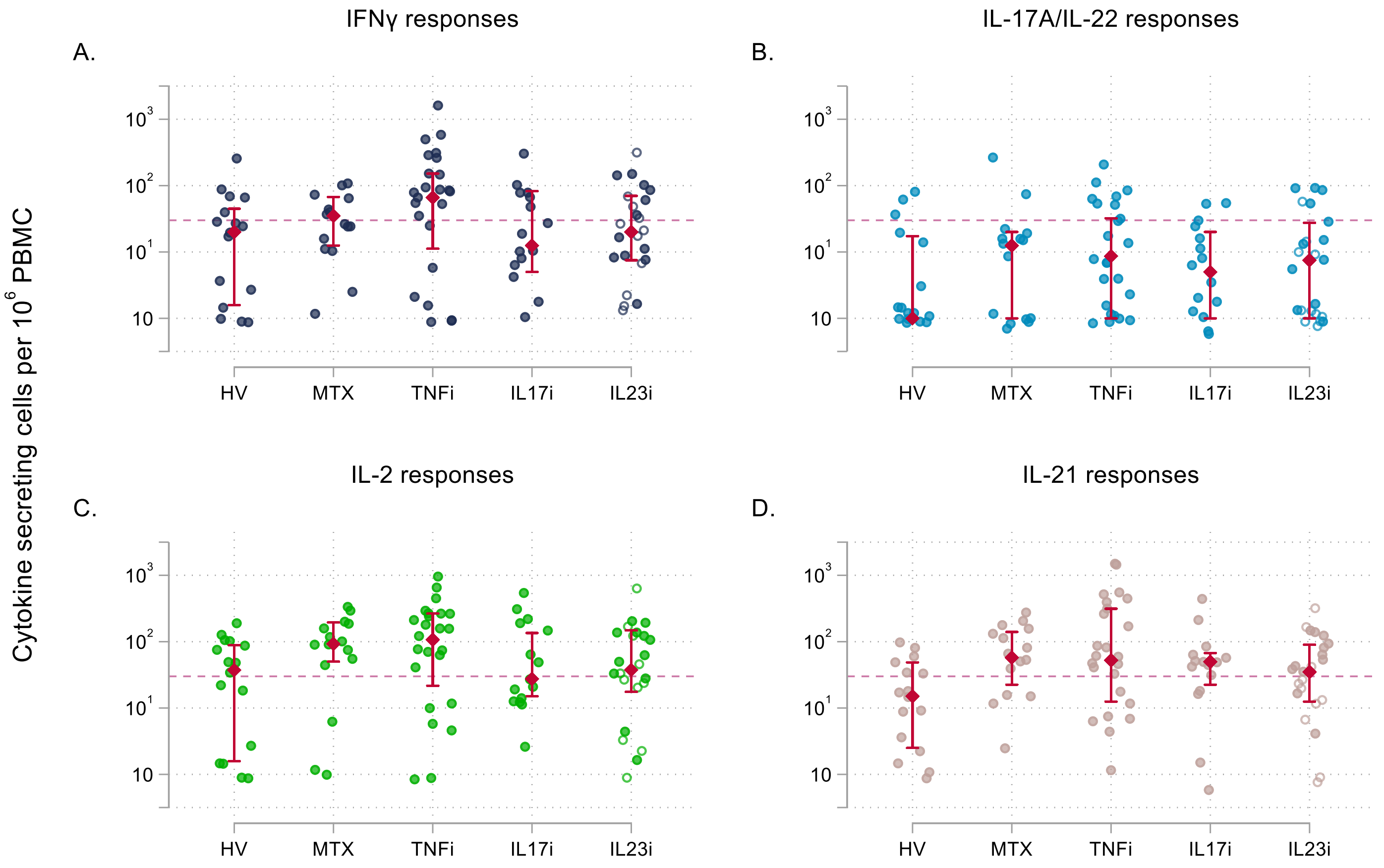

The circles represent individual values. The red diamonds and range lines indicate the median and interquartile range (IQR). In the IL23i group, filled circles represent participants receiving IL-23p19 inhibitors and hollow circles represent participants receiving IL-23p40/IL-12 inhibition. Horizontal line indicates T cell response threshold. HV, healthy volunteers; MTX, methotrexate; TNFi, TNF inhibitors; IL17i, IL-17 inhibitors; IL23i, IL-23 inhibitors.

Figure 4E. Correlations between humoral and immunogenicity assays.

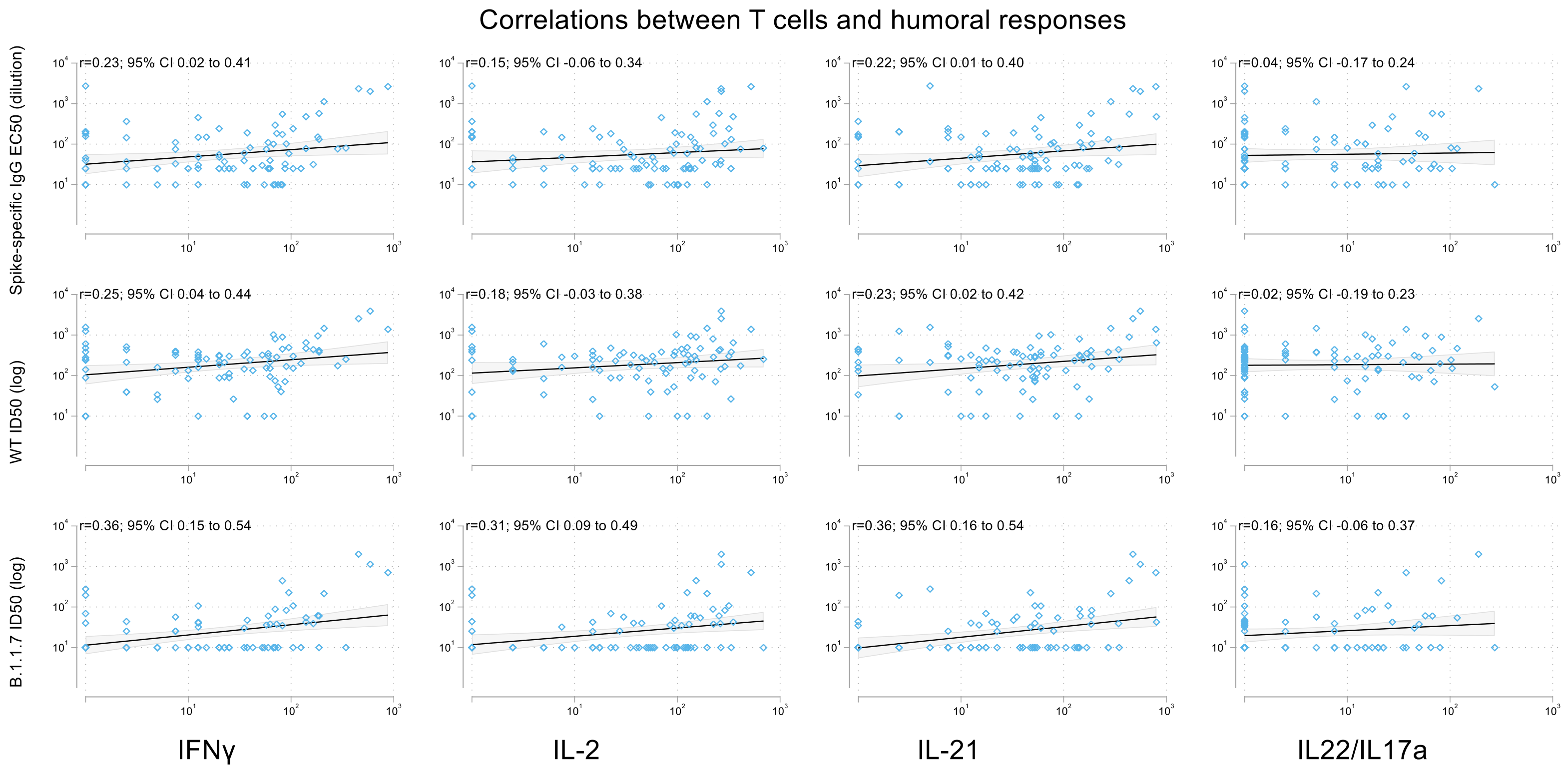

The diamonds represent individual values. n: total number individuals; r: rho by Pearson's correlation; 95% CI: 95% confidence interval.

Appendix Figure 5: **Humoral and cellular immunogenicity of the first dose of COVID-19 vaccine BNT162b2 in patients with prior SARS-CoV-2 infection receiving therapeutic immunosuppression, in comparison to the (infection-naive) whole study cohort**

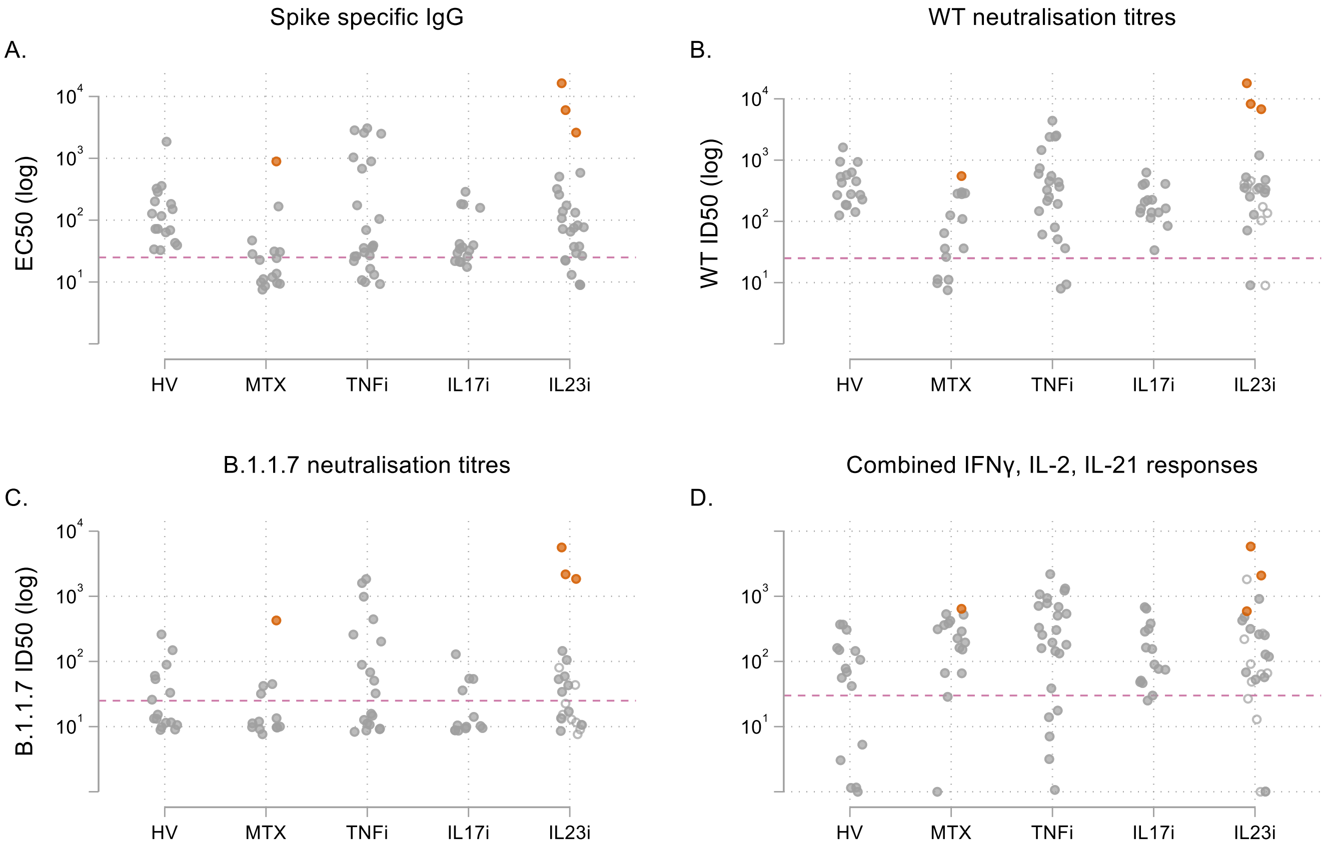

A. Serologic immune responses to the first dose of COVID-19 vaccine BNT162b2. B. Neutralization titres against WT SARS-CoV-2. C. Neutralization titres against B.1.1.7 SARS-CoV-2 variant. D. Total T cell response, as determined by IFNγ and/or IL-2 and/or IL-21 cellular responses.
The circles represent individual values. Orange circles represent participants with prior SARS-CoV-2 infection. Grey circles represent the whole study (infection-naive) cohort. In the IL23i group, filled grey circles represent infection-naive participants receiving IL-23p19 inhibitors and hollow grey circles represent participants receiving IL-23p40/IL-12 inhibition. Horizontal line indicates seroconversion, neutralising activity or T cell response thresholds, as appropriate. HV, healthy volunteers; MTX, methotrexate; TNFi, TNF inhibitors; IL17i, IL-17 inhibitors; IL23i, IL-23 inhibitors.
